# Current Concepts in Pathogenesis and Conservative Management of Supraspinous Tendinopathies Using Shockwave Therapy—A Narrative Review of the Literature

**DOI:** 10.3390/biomedicines13092253

**Published:** 2025-09-12

**Authors:** Roxana Nartea, Ioana Ghiorghiu, Maria-Delia Alexe, Gavril Lucian Gheorghievici, Brindusa Ilinca Mitoiu

**Affiliations:** 1 Rehabilitation Department, “Universitatea de Medicină și Farmacie Carol Davila”, 050474 Bucharest, Romania; 2 National Institute of Rehabilitation Physical Medicine and Balneoclimatology, 030167 Bucharest, Romania

**Keywords:** supraspinous tendinopathy, conservative treatment, shockwave, rehabilitation

## Abstract

The supraspinatus has been well studied in recent years, since the rotator cuff muscle is most commonly damaged, affecting the quality of life for patients with tendinopathy. Despite this tiny but crucial muscle being well-described, there is still much left to learn and discover, especially because chronic supraspinatus tendinopathy is a common pain syndrome that causes functional and labor disabilities in the population. The present narrative review, including systematic elements from biomechanical and clinical studies, highlights the role of conservative treatment using shockwave therapy. **Objectives**: We set out to perform a complex analysis of the literature on this topic to identify possible pathophysiological mechanisms that can be used to establish proper therapeutic management, focused specifically on conservative treatment using shockwave therapy. **Methods**: We researched the medical literature, including clinical studies that met the criteria of being published after 2014. In this review, we described the mechanisms and histological changes in tendinopathy to illustrate the biological effects of shockwave therapy and performed a literature review on its therapeutic effects. **Conclusions**: The multifaced pathophysiology of supraspinous tendinopathies compromised the healing responses. Recent findings highlight the value of a thorough diagnostic and treatment strategy, with shockwave therapy (ESWT) as a conservative treatment option. According to available data, shockwave therapy has demonstrated significant improvement in function, improving participants’ discomfort, quality of life, and pain relief. It can also be combined with isometric exercise. Given the substantial risk of bias and heterogeneity, our findings should be interpreted cautiously. The need for more research to optimize parameters and provide standardized clinical guidelines is highlighted by the variety of treatment procedures. Improving outcomes in patients with supraspinous tendinopathies will require a better comprehension of the pathology, and individualized treatment needs to be further investigated.

## 1. Introduction

Shoulder pain, experienced as acute or chronic, can be caused by supraspinatus tendinitis. Triggers may include carrying heavy loads in front of and away from the body, injuries from throwing with the hands, or aggressive usage of exercise equipment. Younger people, due to overuse or improper use of the shoulder joint, develop acute supraspinatus tendinitis, which frequently does not receive proper treatment and rest. Older people are more likely to develop chronic supraspinatus tendinitis, and it typically manifests more gradually or subtly, without any identifiable prior trauma [1,2].

The severe and persistent discomfort caused by supraspinatus tendinitis causes intense, ongoing pain that frequently interferes with sleep and affects around 19–25% of the general population during their lifetimes. The primary site of discomfort is the deltoid muscles. It is moderate to severe and could cause the affected shoulder to gradually lose its range of motion. The patient often wakes up during the night while turning over onto the afflicted shoulder [3,4,5]. There appears to be consensus on the need for early diagnosis to facilitate timely surgical intervention and improve patient outcomes, even though the terms “acute” and “traumatic”, used to classify rotator cuff tears, have been the subject of considerable debate in the literature [6,7].

The supraspinatus has been well studied in recent years, since the rotator cuff muscle is most commonly damaged. Despite its small dimensions, this muscle has been well-described, but there is still much left to learn and discover, especially because chronic supraspinatus tendinopathy is a common pain syndrome that causes functional and labor disabilities in the population.

The most common alterations caused by tendinopathy include neovascularization, disordered fibrils, and increased tendon thickness. All of the above usually appear after repetitive microtrauma, which is typically brought on by loading activities [8,9]. A wide range of therapies were used as conservative treatments in tendinopathies, focused on developing an effective solution for all the symptoms mentioned above.

The actual management protocol for supraspinatus tendinitis includes physical rest, drugs such as anti-inflammatories, local injections with corticosteroid, and conservative rehabilitation procedures such as physiotherapy. Many studies in the literature have examined the impact of physiotherapy procedures (TENS, ultrasound, laser), and more modern ones, such as shockwave therapy, in particular, on musculoskeletal disorders. We want to provide a comprehensive overview and summarize the advantages of using shockwave therapy (ESWT) for the supraspinatus muscles due to the lack of specificity in the literature regarding the limits and the ability of practitioners to use ESWT [7,8,9,10].

After reviewing treatment protocols in the literature, we intend to highlight the need for a more comprehensive understanding and establish that the optimal number of sessions, intensity, pulses, and treatment intervals differ when treating shoulder tendinopathy. This narrative review aims to synthesize the existing information and set current directions for the conservative treatment of tendinopathy, focused on the supraspinatus muscle.

Different types of passive physical therapy are often prescribed in conjunction with medical treatment. However, there are no guidelines for physical therapy in MSK conditions, including shoulder tendinopathies, with practical recommendations graded according to the scientific evidence. In recent years, many studies have shown promising results in improving function, restoring range of motion, reducing pain, and improving ADL in patients with supraspinatus tendinopathy.

This narrative review aimed to

- Briefly overview the current literature on the pathogenesis of supraspinatus tendinopathies;
- Provide the most recent and convincing evidence on the ESWT effects on supraspinatus tendinopathy;
- Compare the importance of using ESWT in different stages of supraspinatus tendinopathy, an essential aspect for future practice and recommendation in the rehabilitation area.

## 2. Materials and Methods

### 2.1. Data Extraction

From January 2024 to July 2024, research was conducted across several significant databases, including PubMed, Scopus, EBSCOhost, Google Scholar, Academic Search Premier, ScienceDirect, and SpringerLink, to develop a narrative review of the actual literature regarding the use of ESWT on shoulder supraspinatus tendinopathy. We used the operators “AND”, “OR”, and “shoulder” OR “rotator cuffs” OR “conservative treatment” OR “Shockwave”, OR “Physical Therapy”; the following essential phrases were added and combined: (“exercise” OR “acute exercise” OR “chronic exercise” OR “training” OR “physical activity” OR “endurance training” OR “resistance training”) for the selection of studies. In addition, relevant studies were found in the full-text publications’ reference lists and by searching related articles and citations in the PubMed database. The authors submitted the article in the International Platform of Registered Systematic Review and Meta-Analysis Protocols with the number INPLASY202480067.

A narrative review with systematic elements (rather than a meta-analysis) was conducted because of the significant heterogeneity of the included studies concerning the type of physical therapy method, participant characteristics, and physical activity level.

### 2.2. Study Selection—Inclusion and Exclusion Criteria

We performed research through the medical literature, including clinical studies that met the criteria of being published after 2014 and having a minimum seven-point score on the PEDro scale (see Table 1 below).

### 2.3. Characteristics of the Included Articles

Two authors (M.-D.A. and R.N.) separately evaluated the titles selected from the abovementioned literature search based on the inclusion and exclusion criteria (over 300 articles). At each step of exclusion, we used the title and abstract of each of the 300 articles first selected, and after that, we performed a full-text screening of the articles remaining, as noted in Table 2. Possibly relevant citations were selected down to the abstract level. When the abstracts indicated that full-text publications should be included, those papers were reviewed (over 190 articles). For each qualifying study, relevant data were gathered, including sample size; participant characteristics; exercise modality, type, and intensity; and physical therapy type and duration. When available, changes in ROM and discomfort were also recorded. Any disagreements among the authors about the selection of studies or the procedure for extracting data were resolved by discussion and consensus.

**Figure 1:**
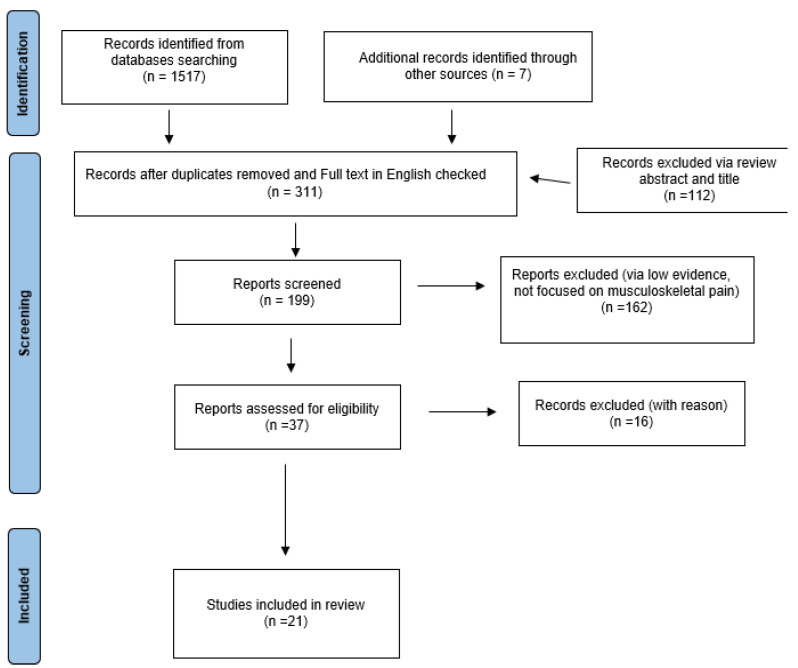
PRISMA table regarding the identification of studies for the research.

Initially, we identified a total of 1517 records, and later, we added 7 more records. Then, we began removing duplicates and performed English language detection, also excluding several articles (via review of title and abstract), resulting in 311 records. We performed a more detailed assessment of the articles, and we found 21 studies that met all our criteria for the final stage, excluding reports with minimal evidence, not focused on musculoskeletal pain of the shoulder, or with no outcome measure. (Figure 1).

In order to ensure the best validity and reliability of the study, the two authors assessed the reviews, and all discrepancies were discussed with another reviewer (B.I.M.). We used a quality assessment for our included studies, evaluating the methodology of the study and the risk of bias; in this way, we could easily examine the results in context with the strength of the evidence.

### 2.4. Risk of Bias

We performed the risk of bias analysis for each type of study, as follows:

- For RCTs/prospective-randomized clinical trials, we used RoB 2 (Cochrane) as an instrument, evaluating the type of randomization, allocation of participants to the study, blinding, loss to follow-up, and selective reporting.
- For observational/retrospective studies, we used ROBINS-I, and we analyzed participant selection, confounding, intervention classification, loss, and selective reporting.
- For narrative reviews, we calculated the intrinsic high risk of bias based on literature selection, type of selective reporting, and presence or absence of standardization.
- For meta-analyses/systematic reviews, we used AMSTAR 2, and we evaluated the inclusion criteria, search strategy, quality assessment of included studies, statistical analysis, and error reporting—see Table 3 below.

Based on the risk of bias table for these articles, we can draw the following conclusions regarding the effectiveness of extracorporeal shockwave therapy (ESWT) in supraspinatus tendinopathy:

1. Quality of evidence

- Most studies are narrative reviews and observational studies (retrospective or mechanistic), which have a high risk of bias. This means that their results should be interpreted with caution, as literature selection and selective reporting may exaggerate the positive effects of ESWT.
- The available RCTs [20,22,26] display a moderate risk of bias, meaning that the data are more robust, but details such as, blinding or loss to follow-up may influence the results.

2. Results from meta-analyses

- Systematic reviews and meta-analyses [13,23,28] have evaluated RCTs and provide more reliable evidence.
- However, the high heterogeneity between studies (type of ESWT—radial vs. focal, energy, frequency, combinations with physiotherapy) and the variable quality of RCTs lead to a moderate risk of overestimation of effectiveness.

3. Involvement of observational and narrative studies

- Retrospective studies and narrative reviews generally suggest that ESWT reduces pain and improves function, but these conclusions are more susceptible to bias.
- Bibliometric and mechanistic studies [15,17] do not provide direct clinical evidence, but support possible mechanisms of ESWT.

4. Overall conclusion regarding the bias risk

- The efficacy of ESWT in supraspinatus tendinopathy is likely proven, especially for reducing pain and improving function, but
  - Strong clinical evidence is limited to only a few RCTs, which carry a moderate risk of bias.
  - Narrative reviews and observational studies may exaggerate effects.
  - Heterogeneity of the ESWT protocol limits the generalizability of the results.

Practical interpretation: ESWT can be considered a therapeutic option, but the level of evidence is moderate, and recommendations should be individualized. Ideally, better-controlled and standardized RCTs are recommended to confirm efficacy and safety.

## 3. Histological Aspects Regarding Tendon Structure in Pathology

This section aims to present a general overview regarding the structural histological aspects of the tendon in pathological conditions. We based this section on the current literature database, including the 21 studies included in our review.

The supraspinatus muscle is an important muscle in the shoulder joint that assists in stabilizing the shoulder by reducing the inferior gravitational stresses that the weight of the upper limb exerts downward on the shoulder joint. The supraspinatus muscle abducts the arm at the shoulder by firmly pressing the head of the humerus against the glenoid fossa [4]. The suprascapular nerve, which is made up of fibers from the superior trunk of the brachial plexus, innervates the supraspinatus muscle [1,4]. The muscle inserts into the top facet of the greater tuberosity of the humerus after emerging from the supraspinous fossa of the scapula. The tendon’s inferior part is closely connected to the joint capsule when the muscle crosses the superior face of the shoulder joint beneath the acromion. The musculotendinous unit is susceptible to impingement as it passes beneath the acromion. A posterior and an anterior muscle belly are present. The anterior muscle belly is larger and passes through a narrower tendon region [1,2,4]. Since the anterior tendon serves as the main contractile unit, rotator cuff tendon repairs should, if possible, include the anterior tendon because its stress is substantially higher than the posterior tendon’s [1,3].

### 3.1. Tendon Structure

Tendons, which connect muscle to bone, are flexible, pliable structures. Their primary job is to transmit the force produced by the muscles to the bone system, which makes movement around a joint easier. As a result, they are relatively inert, rigid structures that can withstand strong stresses. Collagen makes up the majority of tendon tissue and is hierarchically parallel to the tendon’s long axis, giving it a high tensile strength. Various non-collagenous proteins are also present in small amounts in the tendon, yet they play crucial physiological roles [29].

Four structural subunits within the supraspinatus tendon were identified by Fallon et al. after studying the histological morphology of the tendon: (a) the tendon proper, (b) the attachment fibrocartilage (which measures approximately 2.8 cm in length), (c) the rotator cable (an extension of the coracohumeral ligament), and (d) the capsule [30]. During the dissection, they observed that the anterior tendon is a more tubular, “rope-like” structure, and the posterior tendon is a “thin strap-like” formation, which together generate a large point of attachment on the greater tuberosity [30]. The “axis of tension” was discovered to be parallel to the collagen fibers and fascicles, and histological staining revealed a wealth of negatively charged glycosaminoglycans that served as points for the fascicles to move against and readily separate from one another. In the front tendon, compared to the posterior tendon, the fascicular organization of the tendon is properly shifted to a basket-weave pattern of the attachment fibrocartilage [31,32,33].

Histologically, the attachment fibrocartilage region resembled compressible fibrocartilage. As a perpendicular extension of the coracohumeral ligament, the rotator cable is situated between the tendon and the joint capsule and properly runs parallel to the tendon’s axis [32,33,34]. It was discovered that the joint capsule is made up of some thin collagen sheets, with the collagen fiber orientation varying between layers but remaining constant within sheets. Together, the sheets’ collagen fibers are arranged in a variety of ways to form a thin but powerful framework. The attachment fibrocartilage and the capsule are fused at a location just medial to the supraspinatus tendon insertion on the greater tuberosity [31,32].

The inferior part of the tendon fuses with the joint capsule around 1 cm before the attachment to the bone. The supraspinatus tendon and muscle both enter the greater tuberosity of the humerus through an opening below the acromion process. The superior portion of the tendon is joined to the coracohumeral and transverse humeral ligaments. The muscle can move the humerus into abduction in this position and location. Together with the deltoid, the supraspinatus plays a role in the movement of abduction at the beginning and throughout the range of motion. It has also been demonstrated that the supraspinatus makes a minor contribution to the humerus’ lateral rotation [5,32].

The extracellular matrix (ECM) content divides the insertional supraspinatus tendon anatomically into four transitional layered zones: 1. The first zone consists primarily of type I collagen and a modest amount of decorin (proteoglycan associated with collagen, representing a common component of connective tissue and the extracellular matrix). The tendon proper may be thought of as this region. 2. Type II and III collagen make up the majority of the second zone, with trace amounts of type I, IX, and X collagen creating fibrocartilage. 3. Mineralized fibrocartilage made of type II and X collagen, aggrecan (proteoglycan), and other proteins serves as the boundary for the third zone. 4. The effective bone–tendon connection is defined by the collagen fiber orientation in the fourth zone, which is created by type I collagen [35].

### 3.2. Tendon Changes in Tendinopathy

Although some hypotheses have suggested that tendinopathy occurs by inflammation via the separation of collagen fibers and degeneration from recurrent stress leading to microtrauma, more recent research showed that in some cases (chronic Achilles tendon disease), there is no inflammation present [6,36]. The term tendinosis, which is used to describe tendon pain caused by a disjointed healing process taking place in the tendon structure [37], is also used. The use of these terms following a histological investigation has been advised; however, this does not seem appropriate for all individuals with tendon pain [37]. As a result, new theories and research have been developed.

**Figure 2:**
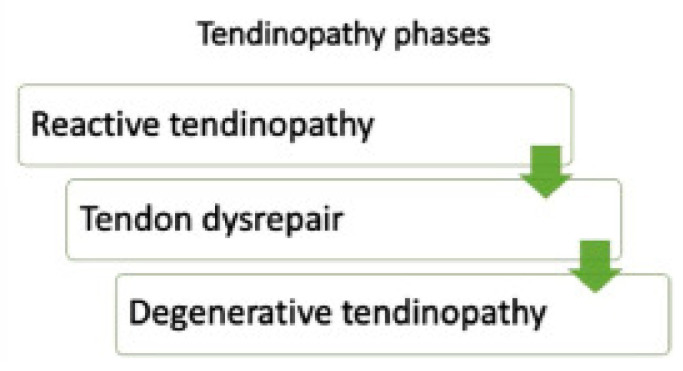
Tendinopathy phases [14].

The tendon continuum is a new approach that Cook and Purdum have suggested for dealing with tendon pain [38,39]. They contend that this continuum comprises three stages. (A) Disorganized collagen with fibers that are thinner than usual, accompanied by a loss of the traditional hierarchical structure. Because normal collagen fibers cannot rip in vivo without significant changes in the non-collagenous matrix, the collagen-tearing hypothesis is one of the oldest and is subject to criticism. The long-lasting properties of typical tendon collagen indicate that collagen tearing and remodeling do not result from frequent, severe loading but rather from early alterations of the collagenous matrix (such as fiber kinking and a “loosening”). As a result, collagen disruption theory-based animal models of tendinopathy (collagenase injection, tendon laceration) have little application to human tendinopathy in vivo.) [38,39,40], (B) A tendon’s response to overuse is inflammation, which is a very complex process [38,41,42,43]. Numerous studies have documented the characteristic inflammatory response in the tendon that occurs when a tendon (and its blood supply) is torn or lacerated [44,45,46,47]; this reaction is characterized by a significant immune cell and tenocyte response that boosts protein synthesis and tendon growth. Despite the presence of inflammatory cells in diseased tendons, the reaction does not appear to constitute a typical inflammatory response [48,49,50]. Overuse tendinopathy has been associated with increases in inflammatory cytokines (e.g., COX-2, PGE-2, interleukin (IL)-6, IL-1β, and transforming growth factor (TGF- β), but the presence of these molecules does not necessarily strengthen the idea that inflammation is the main cause or major contributor to tendon pathology [42,43]. Inflammatory indicators such as IL-1, IL-6, PGE-2, IL-1, vascular endothelial growth factor, COX-2, TGF-α, and β are altered in response to cyclic stressors; tendon cell culture research shows that the local tenocytes are the ones responsible for the expression of those cytokines [42,43,49]. The increase in inflammatory cytokines seen in tendon pathologies may be caused by tendon cell overload signaling (tendons becoming mechanoresponsive), which subsequently induces matrix remodeling, affecting both the synthesis and degradation of proteins [39,49,51,52]. (C) The continuum model’s last stage is characterized by permanent alterations, pockets of cell death, trauma, tenocyte depletion, and general disarray of the cell matrix. According to imaging examinations, the tendon [53,54] seems to display areas of this degeneration strewn about, mixed with healthy sections and areas that are in the disrepair stage. On palpation, the tendon may be thicker, with several nodular portions. Clinically, this tendon is found in older people who continue to struggle with tendinopathy or in younger people who continue to overuse the tendon [39]. Tenocytes are in charge of maintaining the extracellular matrix in response to modifications brought on by outside forces. The tenocyte detects any change at the tendon level and triggers a series of reactions, including cellular activation, increased proteoglycan expression, and changes in the type of collagen. The same paradigm can be used to explain nodulations that develop at the level of the secondary tendon as a result of direct trauma or chronic diseases (enthesis pathology) [38,47]. However, it is still unclear how collagen fiber breakdown or rupture models take into account these properties. (See Figure 2 for tendinopathy phases).

The pathophysiology of tendinopathies has also been explained by several alternative models. To understand the positive response to loaded therapeutic exercises shown in other studies, Littlewood et al. examined the implications of the central nervous system (CNS) [55]. In his study, Coombes offered a model based on integrating pain that included three interconnected elements: (i) the local tendon disease, (ii) modifications to the pain system, and (iii) impairments to the motor system [56]. Lui et al. recently examined the role of oxidative stress in tendinopathy. Reactive oxidative stress (ROS) generation may play a role in the etiology of tendon diseases, according to their review [43].

In conclusion, it is challenging to build a simple and robust model that takes into account all facets and phases of the condition due to the complexity of normal tendon structure, the multifaceted nature and magnitude of the tendon’s response to injury, and the obstacles in developing an experimental model that mimics load-related tendon pathology in humans. Whatever the initial event (overstimulation of resident tenocyte, collagen disruption/tearing, inflammation), tendon pathology is defined by a significant cell response to injury. Due to the complexity of these processes and interconnections, it is improbable that any one model can adequately explain all facets of the etiology of tendon pathology and its connections to pain and function.

### 3.3. Histological Changes Associated with Supraspinatus Tendinopathy

Under the microscope, tendinopathy is differentiated from normal tendons by four major changes, as presented in Table 3: (A) disrupted collagen, in which the collagen fibers are thinner than usual, and the distinctive hierarchical structure is lost; (B) increased ground substance, with a high concentration of glycosaminoglycans; (C) more prominent and numerous tenocytes without their normal, fine spindle shape and with more rounded nuclei; and (D) neovascularization as seen on color and power doppler ultrasound images [57].

The histological changes to the tendon (as it can be seen in Table 4) include decreased fibroblast numbers and rounding; increased proteoglycan, glycosaminoglycan, and water content; hypervascularization; and disordered collagen fibrils. Messenger RNA levels for type I and III collagens, proteoglycans, angiogenic factors, stress and regeneration proteins, and proteolytic enzymes are all elevated at the molecular level. Tendon tears and material fatigue have been proposed as potential damage processes, suggesting that the structure may have one or more “weak links”. Understanding the pathophysiology of tendinopathy requires an understanding of how tendon tissue responds to mechanical loading [51,53,58].

The articular surface of the anterior insertion on the humerus, where the stresses are believed to be the largest, is where lesions of the supraspinatus tendon appear to begin. It has been hypothesized that excessive mechanical pressures at the supraspinatus tendon insertion enhance the rate of collagen synthesis and turnover, which are frequently linked to tendon tears and ruptures [2]. Many intrinsic and extrinsic variables have been proposed as contributors to the development of supraspinatus tendinopathies, even though the etiology is still poorly understood [33].

From the cellular point of view, tenocytes, the most prevalent cell type in the tendons, are in charge of maintaining the health of the tendons by secreting ECM and collagen [59]. They are extended in the tensile-load-bearing portions of their tendon mid-substance and have a spherical form in the fibrocartilaginous regions. In addition, smooth muscle cells, endothelial cells, and synovial-like cells are linked to blood vessels. Some alterations seem to occur frequently as tendinopathy progresses. Hypoxia, an increase in tiny nerve density, and an increase in nociceptive chemicals and neurotransmitters, including substance P and glutamate, are all common. Tenocytes typically develop a fibrochondrogenic phenotype, lose their natural shape, become necrotic/apoptotic, and multiply. Particularly, Scott et al. discovered that after injury, supraspinatus tenocytes became more chondroid and displayed enhanced proliferation in an animal model [42,47,60]. The amount of collagen in the ECM is decreasing, the type III/type I collagen ratio is rising, the collagen fibers are becoming thinner, and hyaline degeneration, chondroid metaplasia, and lipid infiltration are all present. Often, hyaluronan, chondroitin, and dermatan sulfate are also present in higher amounts. Riley et al. have demonstrated that decreased gelatinase (MMP2) and stromelysin (MMP3), as well as enhanced collagenase (MMP1) activity, are all connected with RC tendinopathy. This could be an adaptive reaction to mechanical pressures, pointing to a high level of collagen turnover [61].

In regards to whether inflammatory cells are present or absent in tendinopathy, there is still some disagreement. While some researchers claim that degenerating tendons do not include inflammatory cells, others maintain that the emergence of tendinopathy is accompanied by the presence of inflammatory cytokines [43,59,62]. In contrast to the dazzling white-hued, robust fibroelastic normal tendon, tendinopathic tendons typically have a thin, mushy, and fragile crux that is irregularly colored gray or brown [41,63,64].

## 4. Risk Factors Associated with Tendinopathy

**Figure 3:**
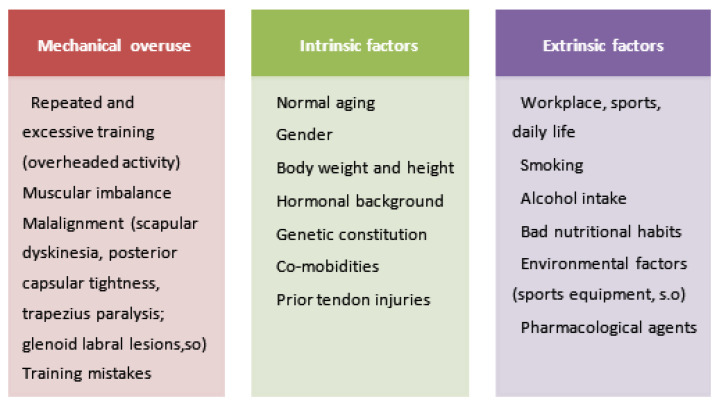
The pathogenesis of tendinopathy is shown schematically. The continual onset of tendinopathy is thought to be caused by a variety of risk factors, such as mechanical overuse and intrinsic and extrinsic causes [1,10,12].

Many different etiologic factors seem to contribute to tendon diseases, as is summarized in Figure 3. Lesions of the supraspinatus tendon appear to begin at the articular surface of the anterior insertion on the humerus, which is where the stresses are believed to be the greatest [1]. It has been hypothesized that excessive mechanical pressures at the supraspinatus tendon insertion enhance the rate of collagen synthesis and turnover, which are frequently linked to tendon rips and ruptures [65]. Many intrinsic and extrinsic variables have been proposed as fundamentals of supraspinatus tendinopathies, even though their etiology is still poorly known [33].

One explanation for the different types of tendinopathy is that different triggers may start the process, which progressively leads to distorted cell–cell and cell–matrix communication. As a result, the cell and matrix organization of the tendon tissue is lost, inevitably resulting in tendon rupture. In this process, endogenous tendon stem cells and progenitor cells can respond abnormally to signals and choose entry into alternative cell outcomes, which results in tendinous fattening and calcification. Exogenous lineage cells can also be activated, causing vascular and neuronal ingrowth, inflammation, and pain [59]. There are three major hypotheses regarding tendinopathies resulting from altered mechanical loading: (1) mechanical overuse (through the matrix); (2) vascularization supply (through exogenous cells); (3) tendon decline due to aging (through endogenous cells). All three of these triggers most certainly cross-talk and cross-react; however, thorough investigations of each have not yet been conducted systematically [1,43]. As a result of the lack of tools to identify the initial changes, it has been impossible to determine the exact rate of illness progression up to this point. Additionally, because it is a multidimensional illness, the rate of progression may vary, for instance, between calcifying tendinopathy and fattening tendinopathy.

Genetics plays a major role in the majority of intrinsic risk factors for common tendon and ligament problems [66]. For example, the heritability of flexibility is believed to range between 64 and 70% [67,68]. Siblings of patients rotator cuff issues exhibit a five times higher risk of developing rotator cuff problems, according to familial research [69]. Moreover, many seropositive and seronegative rheumatological illnesses (including gout and ankylosing spondylitis), Ehlers–Danlos syndrome, and other endocrine and metabolic disorders all display a hereditary component that raises the risk of tendon pathology [70]. Nevertheless, extrinsic factors (for example, training load) interact with a person’s genetic background and other intrinsic factors to create a large number of sports injuries [71].

Aging has been linked to tendinopathy and has been demonstrated to negatively affect tendon characteristics, especially after the age of forty [72]. The suppleness of tendons and their resistance to tensile loads tend to decline with aging [39,73]. Moreover, Kumanagai et al. demonstrated in a study with participants aged 52 and older that the aging supraspinatus tendon exhibits calcifications, changes in fibrovascular proliferation, a decrease in total glycosaminoglycan content, and changes in proteoglycan content [74].

One of the main causes of supraspinatus tendinopathy is believed to be impingement syndrome, which is caused by the mechanical compression of the rotator cuff tendons and is influenced by the shape, angle, and existence of acromion–clavicular spurs [75,76]. Reduced sub-acromial space and shoulder impingement may also result from posterior capsule tension, which may force the humeral head to migrate anteriorly rather than superiorly [34,77]. Changes in scapular kinematics have been connected to the supraspinatus and rotator cuff tendinopathy, as well as to strength deficiencies and postural deviations, though this connection is still up for debate [4,77,78].

The risk of developing tendinopathy and its poor prognosis have been linked to obesity and its comorbidities, such as hyperglycemia, diabetes, dyslipidemia, and hypercholesterolemia [79,80,81]. ROS (reactive oxygen species) production and consequently, oxidative stress, increases in parallel with adipocyte hypertrophy and adipose tissue hyperplasia, promoting tissue inflammation [62]. In obesity, the ratio of pro- to anti-inflammatory adipokines is disrupted, which favors systemic low-grade inflammation. There is strong evidence that inflammation brought on by obesity plays a causative role in the emergence of several obesity-related illnesses, including diabetes mellitus, cardiovascular disease, and cancer [79,80,81,82]. Recent research has demonstrated that several adipokines that are dysregulated in obesity can cause heterotopic ossification in tendons via the mTOR (mechanistic target of rapamycin) pathway [62,83]. Type 2 diabetes and hyperglycemia are caused by obesity. The cellular response to oxidative stress is significantly influenced by extracellular glucose. If glucose levels are high, oxidative stress that is typically catabolic becomes pathogenic [43,62]. High glucose concentration led to cellular death, while low glucose concentration encouraged differentiation of H_2_O_2_-stimulated tenocytes [84].

The patient’s age, gender, mobility, and general state of health may also have an impact on the healing process. All things considered, it can be challenging to determine the “one size fits all” or averaged kinetics of the complete tendinopathy process for human populations. Numerous biological characteristics, lifestyle-related factors, pharmaceutical drugs, and other variables are thought to have a significant impact on the development of chronic tendon diseases. In Figure 3, we separated all these factors into intrinsic (acting from within the body) and extrinsic (acting on the body) etiological components, which are mechanical overuse- and load-related triggers.

In addition to mechanical challenges, which are considered to be one of the main early causes of tendinopathy, several systemic (intrinsic), highly individualized factors also appear to have an important bearing on tendinopathy. Age and aging, gender, height and weight, dominant arm-affected genetics, hormonal background (such as menopause, pregnancy), pre-existing disorders (such as obesity, hypercholesterolemia, diabetes mellitus, adiposity, and chronic gouty arthritis), and prior tendon injuries are thus ranked to influence the biological aspect of tendon pathogenesis by lowering the tendon’s ability to tolerate load and by modulating repair responses [54,61,85,86,87].

Several extrinsic factors, including environmental factors, are responsible for increased stress on the tendon, including active daily life and professional physically demanding activity, poor nutrition, alcohol abuse, pharmacological agents (e.g., fluoroquinolone and quinolone drugs, corticosteroids, statins, contraceptive medication, and hormone replacement therapy) [59,87,88,89,90,91].

## 5. Biological and Therapeutic Effects of Conservative Treatment Using ESWT

The supraspinatus has been extensively investigated, since the rotator cuff muscle is most frequently injured. Though this small, but important, muscle has been well documented, much remains to be discovered and learned, particularly since chronic supraspinatus tendinopathy is a prevalent pain syndrome that impairs function and movement in the general population. The conservative treatment of tendinopathies is a must for patients who wish to regain a normal life. We focused our investigation on ESWT, since this component piqued our interest in conducting an actual research study of the literature on conservative treatment.

### 5.1. Shockwave Therapy—Mechanism of Action

For musculoskeletal disorders, shockwave therapy represents an efficient and non-invasive conservative treatment. To alleviate pain and promote tissue repair, ESWT releases growth factor, activates stem cells, promotes anti-inflammatory qualities, and increases blood flow [23]. It employs acoustic waves that produce microtrauma on the applied tissues, which stimulates the healing process of the body, promoting local regeneration. The specific biological changes and therapeutic effects generated by shockwave therapy are listed in Table 5 [11,18,92,93,94,95,96,97,98,99,100,101,102,103].

Extracorporeal shockwave therapy was first used as a noninvasive medical treatment for kidney stones in 1980. Since that time, the area of use has revolutionized to applications for musculoskeletal pathology, the end final stage of ischemic heart disease, prostatitis, neurological pathologies, and wound healing [17,18,95,104,105,106]. General benefits of extracorporeal shockwave therapy involve its proven ability to alleviate pain and enhance functional abitility in a variety of musculoskeletal disorders [9,107].

Cellular mechanisms that transform mechanical stimuli into biochemical signals are known as mechanotransducers. Increased intracellular tension, cellular adhesion, and migration are examples of short-term responses. It is believed that several overlapping and cross-talking signaling pathways can mediate the long-term effect of ESWT [101,102,108].

The damaged area should receive shockwaves. To locate the area to be treated, palpatory methods or ultrasound localization may be performed. It seems that both choices are equally advantageous [109].

After reading application protocols throughout the literature, we found that the intensity, pulses, and number of sessions used for shoulder tendinopathy vary [110,111].

The protocols for rotator cuff (including supraspinous muscle) tendinopathy for ESWT vary in the literature, as noted in Table 6 below [18,19,20,21,112].

In summary, shockwaves reduce pain, aid in tissue repair, and boost bone growth. Nevertheless, we know little about the physiological process by which they produce all these effects. Furthermore, it is quite challenging to determine the number of sessions, the intervals, and the intensity at which ESWT should be applied, considering the wide range of protocols found in the literature. Therefore, additional studies need to present clear methodologies and results to establish guidelines in this sense [11].

### 5.2. Shockwave Therapy—Side Effects and Contraindications

For the best individualized treatment of our patients, we must first ensure that we use shockwaves safely in order to ensure the best results. In order to reduce risks, next we will list the main contraindications.

Contraindications of using shockwave therapy [11,18,113]:

- Coagulation disorders;
- Conditions that promote bleeding;
- Neurovascular pathologies;
- Infections;
- Tumors;
- Rheumatoid arthritis;
- Growth plate issues;
- Pregnancy.

Shockwave therapy is widely considered as a safe, non-invasive option for musculoskeletal disorders. As for any therapeutic procedure, in the literature, the studies also mention side effects after ESWT, including pain, local redness, local swelling, mild local inflammation, and erythema on the skin [18]. In general, these side effects go away in a few days. Also, more important side effects, such as soft tissue lesions or tendon rupture, are noted in the literature. In order to reduce the percentage of side effects, especially the serious types, the procedure must be performed by an experienced and well-trained professional who knows and respects the guidelines.

## 6. Summary and Future Scope

This narrative review provides an in-depth overview of some of the most recent studies on supraspinous tendinopathy, highlighting tissue damage and the advantages of conservative treatment with shockwave therapy. Numerous tendinopathies of the lower and upper limbs demonstrate the positive effects of ESWT, pointing to the benefits of this conservative procedure [11,23,114].

**Figure 4:**
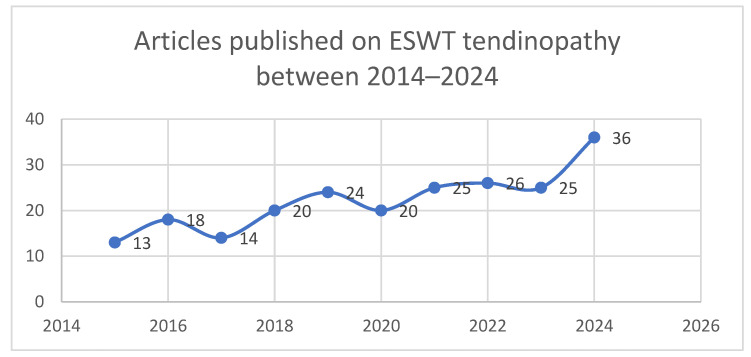
Articles published on ESWT tendinopathy between 2014–2024.

As can be seen in Figure 4 below, in recent years, the literature has revealed a noticeable spike in interest in tendinopathy due to its impact on the quality of life of patients. A conservative approach to treatment by integrating ESWT, which has gained attention in recent years, indicates a bright future for research and the integration of therapies in rehabilitation.

We have reviewed the 21 articles included in the study to provide a broad perspective on the use of shockwaves and their promising conservative treatment, both for the histological and clinical stage of supraspinatous tendinopathy. The methodological protocol of ESWT varies significantly in the literature.

**Figure 5:**
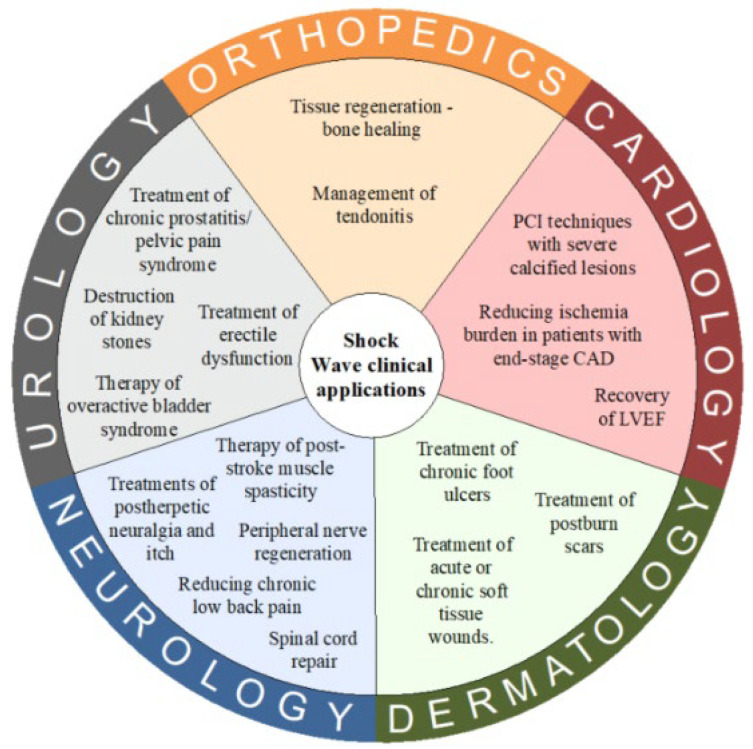
Clinical use of shockwave therapy [105].

We focused our review on the benefits produced in the musculoskeletal field, especially for shoulder pathologies. The clinical applications of ESWT can be clearly observed in the paper by Rola et al., published in *Biomedicines* in 2022, as shown in Figure 5 below.

Due to the confirmed efficacy of ESWT in multiple branches of medicine, it can be used as an adjunct or a standalone therapy [114,115,116]. For tendinopathy of the shoulder, ESWT has demonstrated significant improvement of function, improving participants’ discomfort, quality of life, and pain, especially in combination with isometric strength exercises [12,22]. It is increasingly being used for tendinopathy treatment for both upper and lower limb tendinopathies [10].

ESWT represents an acoustic disturbances that travel through a material carrying energy, with two stages of shockwaves, including a near-instantaneous jump with a tiny pulse width at −6 dB and a high peak positive pressure, sometimes as high as 100 MPa (500 bar) but more frequently between 50 and 80 MPa [105]. The therapeutic use of ESWT is based on the reduction in inflammation, neovascularization, and tissue regeneration by reducing calcium deposits [16,19,106]. Due to all these effects, ESWT is applied in different branches of medicine.

Majidi L. et al, in their meta-analysis published in April 2024, studied many investigations from the literature, evaluating the impact of ESWT used alone or in combination with alternative treatments (other physiotherapy procedures), demonstrating its impact on reducing pain for different types of tendinopathy, using lower intensities and for a long duration of treatment [23].

As Katz NB et al. pointed out in their article, ESWT may also impact estrogen levels in the body, but ultimately, it yields similar effects on both sexes, female and male [117].

A study published by Shahabi points out the need for more rigorous study designs by highlighting a critically low protocol evaluation of the effect of ESWT on tendinopathies [8].

In their review in 2020, Simplicio C et al. mentioned and discussed the efficacy of radial and focused ESWT on many musculoskeletal disorders, including tendinopathy, and they suggest that for severe patients, the results of therapy indicate a low success rate [16].

In their narrative review published in 2018, Reilly et al. pointed out that in the literature, after performing a blinded placebo ESWT treatment, the evidence of ESWT treatment represents a reasonable treatment for musculoskeletal conditions, with low bias risk [18].

In 2018, a retrospective study was conducted by Chou Wen-Yi et al. with 36 athletic and non-athletic patients, divided into two groups, and presented a rate of satisfaction of 53.8% and 52.1%, respectively [19].

In the literature, there are also articles that point out the efficacy of ESWT combined with other conservative procedures, such as kinetotherapy. For example, Frassanito et al. published a randomized controlled trial in 2018, pointing to the significant improvement in both groups during the follow-up and a better improvement in pain on the VAS scale at the end of follow-up (*p* = 0.02) for ESWT combined with KT [20].

Numerous research studies that we reviewed demonstrated a notable improvement in shoulder functional status and pain reduction on the VAS scale, as mentioned by Moya D et al. (2015), Carlisi E. et al. (2018), Majidi L. et al. (2024), and Su X. et al. [21,22,23,24]

The findings from the literature we evaluated for the use of shockwaves for tendinopathies reinforce the benefits of conservative treatment. Also, ESWT combined with other physiotherapy procedures or kinetotherapy reduces pain significantly better than ESWT used alone.

ESWT is now a part of the multimodal, individualized treatment plan in the clinical practice of supraspinous tendinopathies, specifically for patients who do not respond to conservative approaches. Using concepts from the current literature regarding the mechanism of ESWT and its application to tendinopathy disease, we provided a broad overview of studies on the use of shockwaves as a treatment for supraspinous tendinopathy.

In conclusion, even if shockwave therapy displays therapeutic potential, cautious optimism should be maintained about its use, and more randomized controlled trials are needed to confirm its therapeutic efficacy in order to improve its indications, parameters, and use in musculoskeletal disorders.

### 6.1. Strengths

All the studies included present specific methodologies regarding shockwave therapy and include quantitative data with reliable, precise, and consistent information.

### 6.2. Limitations

The present paper presents a limitation that may have influenced our conclusion. Initially, we eliminated several studies based on their availability and publishing status, and we might have overlooked pertinent studies that were published in a language other than English. The absence of a rigorous methodology, transparent reporting of results, and a sufficient sample size may have impacted the review’s overall quality. Also, we have limited the quantitative synthesis of our results

## 7. Conclusions

Examining papers from the past 10 years, this review looks at how the supraspinous muscle changes in rotator cuff disease and also intends to point out how conservative rehabilitation treatment, including new procedures such as extracorporeal shockwave therapy, becomes a common substitute for conventional surgical methods, yielding good results and increasing the patient’s quality of life [118].

The multifaceted pathophysiology of supraspinous tendinopathies, including degenerative alterations, mechanical overload, and compromised healing responses, makes them a complex clinical entity. Recent findings highlight the value of a thorough diagnostic and treatment strategy, with shockwave therapy (ESWT) as a conservative treatment option. According to available data, shockwave therapy has demonstrated significant improvement in function, improving participants’ discomfort, quality of life, and pain relief. It can also be combined with isometric strength exercises. Given the substantial risk of bias and heterogeneity, our findings should be interpreted cautiously. The need for more research to optimize parameters and provide standardized clinical guidelines is highlighted by the variety of treatment procedures. Improving patient outcomes for supraspinous tendinopathies will require a better comprehension of the pathology, and individualized treatment needs to be further investigated. This research supports the necessity for additional long-term monitoring to improve treatment protocols and draw solid conclusions.

## Figures and Tables

**Table 1:** Study selection—inclusion and exclusion criteria.

Assessment Inclusion Criteria:	Exclusion Criteria:
Part 1—All criteria must be fulfilled: Written in English and published in a peer-reviewed journal; Included individuals of any gender, age, weight status, level of physical fitness, and health; Used physical therapy as a standalone intervention or in combination with other physical methods; Applied physical therapy of varying kinds, intensities, and durations; Included at least two measurements (pre- and post-exercise/training) of pain and ROM. Part 2—Assessment criteria of clinical trials included in PEDro.	Reviews, case reports, comments, opinions, or editorials; Applied an intervention without any physical exercise; Did not provide information about the type, intensity, frequency, or duration of the physical therapy applied; Local surgical interventions; Involved animals.

**Table 2:** Characteristics of the included articles.

STUDY (Reference)	Year of Publication	Participant Pathology	Procedure	Number of Studies Included	Conclusion of the Review
Shahabi S. et al. [8]	2024	Tendinopathy—shoulder, rotator cuff with calcification, lower limb	ESWT	18 studies	ESWT was effective in reducing pain and increasing ROM.
Fatima A. et al. [9]	2021	Rotator cuff tendinopathy	ESWT	11 studies	ESWT has no consensus compared to traditional rehabilitation.
Burton I. et al. [10]	2021	Tendinopathy	ESWT combined with exercise	26 studies	Combined ESWT and exercise present a greater reduction in pain and superior ROM recovery.
Corte-Rodriguez H. et al. [11]	2023	Tendinopathy	ESWT	98 studies	ESWT is a safe treatment that can be used in association with other physical therapies.
Fatima A. et al. [12]	2022	Tendinopathy of the rotator cuff	ESWT	42 patients	ESWT is an effective procedure for improving pain, functionality, and quality of life.
Xue X. et al. [13]	2024	Rotator cuff tendinopathy	ESWT	16 studies	ESWT can provide pain relief, maintenance of ROM, and functional recovery.
Canosa-Carro L. et al. [14]	2022	Tendinopathy	ESWT and injections (PRP and corticosteroids)	15	ESWT decreases pain and stimulates the regeneration of tendons.
Wuerfuel T. et al. [15]	2022	Musculoskeletal pathologies (including tendinopathies)	ESWT	42 studies	ESWT applied to different tissues provides different responses, reducing pain, and mimicing the effect of capsaicin.
Simplicio C et al. [16]	2020	Musculoskeletal pathologies—tendinopathy	ESWT	27 studies	ESWT reduces pain and calcium deposits, in moderate cases.
Ji H et al. [17]	2023	Tendinopathy	ESWT	276 studies	The development of more studies regarding pulse parameters is suggested.
Reilly J. et al. [18]	2018	Musculoskeletal pathologies—tendinopathy	ESWT	33 studies	ESWT represents a reasonable treatment for reducing pain.
Chou W. et al. [19]	2018	Shoulder tendinopathy	ESWT	36 patients	ESWT is an effective treatment for athletes.
Frassanito P. et al. [20]	2018	Calcific tendinopathy of the shoulder	ESWT and KT	42 patients	ESWT reduced pain, alone and in association with KT.
Moya D. et al. [21]	2015	Shoulder tendinopathy	ESWT	22 articles	ESWT is a strong therapeutic tool for shoulder pathology.
Carlisi E. et al. [22]	2018	Supraspinatus tendinopathy	ESWT	22 patients	ESWT is effective in improving function and reducing pain.
Majidi L. et al. [23]	2024	Tendinopathies	ESWT	45 clinical studies	Reduces pain and tendinopathy in shoulder.
Su X. et al. [24]	2018	Rotator cuff tendinopathy	ESWT	94 patients	ESWT reduces pain on VAS.
Caballero I. et al. [25]	2024	Rotator cuff tendinopathy	ESWT combined with KT	116 patients	Interesting subject, further studies at work.
Elgendy M. et al. [26]	2024	Tendinopathies—upper and lower limbs	ESWT	25 studies	ESWT presents better results in treating the lower limb than the upper limb.
Santilli G et al. [27]	2024	Supraspinous tendinopathy	ESWT	207 patients	ESWT is a safe and effective therapy for supraspinous tendinopathy, with the best results with combined procedures.
Kuo S et al. [28]	2024	Rotator cuff tendinopathy	ESWT and PRP	55 patients	ESWT combined with the PRP procedure provides a better response.

**Table 3:** Risk of BIAS.

No.	Reference	Study Type	Bias Assessment Tool	Risk of Bias (Summary)	Observations
[9]	Fatima et al., 2021	Narrative review/synthetic analysis	Qualitative assessment	High	Possible selection bias and selective reporting; lack of standardization of inclusion criteria.
[10]	Burton, 2022	Narrative review	Qualitative assessment	High	Study selection is not systematic; risk of selective reporting.
[11]	Corte-Rodríguez et al., 2023	Narrative review	Qualitative assessment	High	Similar to above, a lack of a predefined protocol.
[12]	Fatima et al., 2022	Interventional clinical study (probably RCT)	RoB 2	Moderate	Randomization and blinding details seem incomplete; low risk of unreported losses.
[13]	Xue et al., 2024	Systematic review + meta-analysis	AMSTAR 2	Moderate	Quality assessment of included studies was presented, but high heterogeneity.
[13]	Xue et al., 2024	Correctional meta-analysis	AMSTAR 2	N/A	Does not add new data, only corrections.
[14]	Canosa-Carro et al., 2022	Narrative review	Qualitative assessment	High	Literature selection bias and selective reporting.
[15]	Wuerfel et al., 2022	Mechanistic/experimental study	Qualitative assessment	Low–Moderate	Controlled trials; low risk of bias but limited clinical relevance.
[16]	Simplicio et al., 2020	Narrative/mechanistic review	Qualitative assessment	High	Same problem: study selection and selective reporting.
[17]	Ji et al., 2023	Bibliometric analysis	Qualitative assessment	Low	Objective data (number of articles, citations); low risk of inherent bias.
[18]	Reilly et al., 2018	Narrative review	Qualitative assessment	High	Subjective study selection, selective reporting.
[19]	Chou, 2018	Retrospective comparative study	ROBINS-I	High	Confounding, retrospective selection, lack of blinding.
[20]	Frassanito et al., 2018	RCT	RoB 2	Moderate	Randomization reported; possible risk of insufficient blinding.
[21]	Moya et al., 2015	Narrative review	Qualitative assessment	High	Literature selection is not systematic.
[22]	Carlisi et al., 2018	Interventional clinical study (probably RCT)	RoB 2	Moderate	Similar to Frassanito, minor risk of performance bias.
[23]	Majidi et al., 2024	Systematic review + meta-analysis (RCT)	AMSTAR 2	Moderate	Quality assessment of studies but heterogeneity.
[24]	Su et al., 2018	Retrospective study	ROBINS-I	High	Lack of randomization, high risk of confounding.
[25]	Santilli et al., 2024	Prospective study with predictive model	ROBINS-I adapted	Moderate	An AI model may introduce overfitting; clear patient selection.
[26]	Kuo et al., 2024	Prospective randomized comparative study	RoB 2	Moderate	Good design; low risk of bias, details of blinding unclear.
[27]	Caballero et al., 2024	RCT	N/A	N/A	Published protocol; actual risk of bias unknown until study completion.
[28]	Elgendy et al., 2024	Systematic review + meta-analysis	AMSTAR 2	Moderate	Quality assessment of included studies: moderate heterogeneity.

**Table 4:** Main histological changes in supraspinatus tendinopathy.

Structure Involved	Main Changes	References
Tendon cells	Tenocytes become rounder	[20,27]
	Increased cellularity	[16,30,35]
	Chondroid metaplasia	[1,24]
	Cellular apoptosis	[25]
Extracellular matrix	Degeneration, fatty infiltration	[36,37]
	Loss of distinctive hierarchical structure	[17,35]
Vascularization	Neovascularization	[21,23]
General aspects	Increased number of small nerves	[33,38]
	Increased number of neurotransmitters	[17,19]
	Presence of inflammatory markers	[30]
	Hypoxia	[20,27,30,39]

**Table 5:** Biological and therapeutic effects of ESWT.

Therapeutic Effects	Biological Modifications	References
Tissue Repair	✓ Increased neovascularization of the tissue ✓ Micro-disruption of poorly vascularized tissue ✓ Neovascularization ✓ Tenocytes proliferation ✓ Control over the division, activation, and proliferation of keratinocytes derived from the scar tissue (antifibrosis) ✓ ATP release-coupled Erk1/2 and p38, and MAPK (mitogen-activated protein kinase) pathways	[11,18,92,93,94,95].
Analgesia	✓ Decreased selective number of unmyelinated nerve fibers ✓ Reduced P substance and expression of calcitonin-related peptide in dorsal root ganglia ✓ Serotonergic system activation	[11,96]
Osteogenesis	✓ Control and promotion of bone regeneration and chondrogenesis via the metabolism of mesenchymal stem cells ✓ Increased expression of Pdia-3, which is connected to calcium homeostasis and the 1α,25-Dihydroxyvitamin D 3 Rapid Membrane Signaling Pathway ✓ Decrease in osteoclast activity and stimulation of the periosteum ✓ Osteogenic transcription factors like hypoxia-inducible factor-1α and vascular endothelial growth factor-A (VEGF-A) stimulate osteoblast development ✓ Increased levels of nitric oxide that promote proliferation and osteoblast development ✓ Stimulates the expression of glycoseaminoglycans and hyaluronic acid early expression ✓ Macrophage activation by stimulating bone marrow stromal cells	[11,95,97,98,99,101,102,103]

**Table 6:** Parameters of ESWT used for supraspinous tendinopathy.

1. Focused ESWT: a. Electrohydraulic: 2.000–6000 shocks; depending on the device, between 0.19 and 0.32 mJ/mm^2^; 1 to 3 sessions. b. Electromagnetic: 2.000–6000 shocks; 0.35 mJ/mm^2^; 2–3 sessions.	2. Radial ESWT: 4.000–6000 shocks, 4–7 bar, 3–5 sessions, depending on the device. Interval between applications: 1–2 weeks. Control at 6, 12, 18,2 4 weeks following treatment. No anesthetic in the area.
